# You Can’t Put Old Wine in New Bottles: The Effect of Newcomers on Coordination in Groups

**DOI:** 10.1371/journal.pone.0055058

**Published:** 2013-01-30

**Authors:** Matthew W. McCarter, Roman M. Sheremeta

**Affiliations:** Argyros School of Business and Economics, Chapman University, Orange, California, United States of America; George Mason University/Krasnow Institute for Advanced Study, United States of America

## Abstract

A common finding in social sciences is that member change hinders group functioning and performance. However, questions remain as to why member change negatively affects group performance and what are some ways to alleviate the negative effects of member change on performance? To answer these questions we conduct an experiment in which we investigate the effect of newcomers on a group’s ability to coordinate efficiently. Participants play a coordination game in a four-person group for the first part of the experiment, and then two members of the group are replaced with new participants, and the newly formed group plays the game for the second part of the experiment. Our results show that the arrival of newcomers decreases trust among group members and this decrease in trust negatively affects group performance. Knowing the performance history of the arriving newcomers mitigates the negative effect of their arrival, but only when newcomers also know the oldtimers performance history. Surprisingly, in groups that performed poorly prior to the newcomers’ arrival, the distrust generated by newcomers is mainly between oldtimers about each other rather than about the newcomers.

## Introduction

The composition of groups is rarely stable [1]. Whether in large collectives such as social movements [2] or in small collectives such as work teams [3], it is often the case that *member change* occurs where existing group members (or oldtimers) are replaced by new members (or newcomers) [4], [5]. Because of the ubiquity of member change in organizational settings, a considerable amount of research has surfaced examining how member change affects group performance. A common finding in social sciences, such as organization science, economics, decision sciences, industrial relations, political science, and anthropology, is that member change hinders group functioning and performance [6], [7], [8], [9], [10]. Of course, other research has found the opposite effect: newcomers enhance group performance. However, these studies are different from what we are investigating in that they are particularly focused on creativity and innovation, whereby new ideas are provided by new members [4], [11]. However, questions that have received less empirical attention are why member change negatively affects group performance and what are some ways to alleviate the negative effects of member change on performance?

Moreland and Levine [12] suggest one reason why member change may negatively impact group performance: member change affects intra-group processes. Dineen and Noe [13] similarly posit that *emergent states,* or “properties of the [group] that are typically dynamic in nature and vary as a function of team context, inputs, processes, and outcomes” [14] are likely to explain the effects of member change on group performance (p. 357). Two emergent states posited and found to shape group performance are task flexibility and group learning [13], [15], [16]. For example, team learning and task flexibility suffer from member change (operationalized as team turnover), and, in turn, lead to reduced team functioning on self managing manufacturing teams [16]. In addition, intra-group trust is a third emergent state posited to explain the effects of member change on group performance [17], [18]. *Trust* is an individual’s “expectations, assumptions or beliefs about the likelihood that another’s future actions will be beneficial, favorable or at least not detrimental” [19] to them (p. 576). However, despite acknowledgment of the importance of trust for understanding group and team effectiveness [18], little empirical research has investigated trust in relation to member change in groups. Van der Vegt and colleagues [16] did investigate whether social integration (which includes the element of trust) mediated member change and performance and found no effect. However, their survey measures of social integration neither asked about trust directly nor used complete scales from published research. Further, this work did not control for whether newcomers were replacing oldtimers. The current research complements this previous field work by isolating how the arrival newcomers (while keeping the size of group constant) affected both trust among group members and how oldtimers and newcomers perceived each other.

The one empirical investigation the authors are aware of germane to the current investigation is found in experimental economics: Weber’s [20] study of the weakest-link game with increasing group sizes. In this research, Weber [20] compared cooperation (or what he termed efficient coordination) rates in a 12-person group to a group that grew from 2 to 12 over a series of rounds. Weber [20] also examined whether growing groups could achieve higher levels of cooperation as a function of whether newcomers (who were waiting to play) knew the performance history of the group before entering. The paper reported that groups that grew from 2 to 12, and that shared its history with newcomers, achieved an average cooperation level higher than either the control condition (where the group started and kept the same 12 individuals for all rounds) or growing groups that did not share their history with newcomers.

The Weber [20] article shows one way member change can occur: newcomers may be added to an existing group – making the group larger. A second way is that newcomers can replace existing members [4]. The current article focuses on situations in which newcomers are replacing existing group members. Focusing on member replacement rather than increases in membership holds constant group size, thus helping us to avoid misattributing our findings to changes in member composition when they may actually be due to changes in group size [21]. We also complement the Weber [20] article by analyzing the role of trust in predicting behavioral responses to member change.

The current research investigates how the arrival of newcomers affects intra-group trust among group members and investigates how the potential negative effects of newcomers can be mitigated. Using a coordination (weakest-link) game in an experimental lab setting, our primary findings show: (1) the arrival of newcomers decrease trust among group members and this decrease in trust negatively affects group performance; and (2) knowing the performance history of the arriving newcomers mitigates the negative effect of their arrival, but only when newcomers also know the oldtimers performance history. Surprisingly, (3) in groups that performed poorly prior to newcomers arriving, the distrust generated by newcomers is between oldtimers about each other rather than about the newcomers.

### Theoretical Background and Hypotheses

Groups are traditionally formed to achieve goals that could not be achieved by an individual acting alone [22]. One goal that groups are often intended to achieve is efficiency in coordinating resources [23]. Our conceptualization of coordination is in line with economics and social psychology research which suggest that groups can coordinate to achieve a variety of performance outcomes, ranging from completely efficient coordination to completely inefficient coordination [15], [24], [25], [26]. For instance, there are many settings – ranging from teams to entire economies – where individuals trying to coordinate may be “trapped” in an equilibrium that is inferior to other equilibria, and thus their coordination is somewhat inefficient [15], [25], [27]. Work teams, for example, can “satisfice” and settle for routines that produce suboptimal outcomes but nevertheless create (some) value for an organization [28]. However, work teams can also “maximize” and create routines that produce optimal outcomes, thus leading to more efficient coordination, or totally fail, leading to completely inefficient coordination. Thus, following the work of experimental economics and social psychology, we view coordination as a performance outcome of groups that ranges from completely efficient coordination to completely inefficient coordination [15], [20], [25], [26].

One factor that may influence a group’s ability to coordinate efficiently is trust. As suggested by Camerer and Knez [29] and Schnake [30], coordination in any organized setting requires trust because the individuals incur the risk of being made a “sucker” by those either undependable or unmotivated to contribute their necessary resources toward achieving the collective’s goal. Thus for coordination to occur “harmoniously”, trust must be present among group members [23]. The uni-dimensional psychological approach to trust formation [31] maintains that when groups form, social uncertainty is high and trust among group members begins low (at a conceptual level of zero). *Social uncertainty* (or strategic risk) is a lack of information about another’s behavioral intentions, values, and abilities [32], [33]. Over time and through repeated interaction, familiarity increases and routines become established among group members [34]. Thus, as individuals interact they are able to evaluate whether group members meet their expectations; whether their values are congruent; and their abilities are compatible [35]. As a consequence, social uncertainty decreases and trust increases [36]. As suggested by McCarter et al. [37], group member interaction can build trust in two ways. First, interaction may occur by verbal communication among group members, where intentions are signaled through spoken word [38]. The second type of interaction is behavioral: group members signal their intentions through action, rather than through cheap talk [39]. Because cheap-talk communication does not guarantee efficient coordination in groups [25], the current research examines trust formation through behavioral signaling.

Increased trust is beneficial for the ability of groups to coordinate efficiently because it reduces the perceived strategic risk associated with any individuals’ contributions to the group. By definition, as trust increases, individuals have positive expectations of the intentions or behavior of others in the group (e.g., their contributions of resources to a collective goal), and they are willing to accept vulnerability based upon this expectation. Thus, when a group of individuals trust one another, and thus have positive expectations about others in the group it is easier for individuals in a group to coordinate effectively because they do not anticipate being made a “sucker” by others and their choices should reflect this.

Whereas time and repeated interaction decrease social uncertainty and increase trust, member change and the presence of newcomers increases social uncertainty within a group [40], and decreases the level of trust among group members [41]. Member change occurs in two forms. First, newcomers may be added to an existing group – making the group larger [20]; and second, newcomers can replace existing members [4]. We focus on situations in which newcomers are replacing existing group members. This boundary condition accomplishes two things. Focusing on member replacement rather than increases in membership holds constant group size, thus helping to avoid misattributing our findings to changes in member composition when they may actually be due to changes in group size [21]. In addition, focusing on member replacement removes an additional explanation for changes in group performance: the potential shrinking of shared benefits occurring when groups grow; i.e., when groups grow, the reward must be divided among more people [9].

When newcomers arrive, existing group members are likely to experience reduced positive expectations about the intentions or behavior of others and be less willing to accept vulnerability based upon these expectations. Existing members may be unsure whether newcomers will understand and follow established routines or share the same values and expectations [13], [42]. In turn, the group’s ability to coordinate should be hindered because effective coordination relies on trust in others [23]. Thus, we posit the following.

**Hypothesis 1:** Group members who experience the arrival of newcomers will engage in less efficient coordination compared to group members who do not experience the arrival of newcomers.

**Hypothesis 2:** Group members who experience the arrival of newcomers will trust their fellow group members less compared to group members who do not experience the arrival of newcomers.

**Hypothesis 3:** Trust will mediate the negative relationship between the presence of newcomers and a group’s ability to coordinate more efficiently.

One way that newcomer effects may be mitigated is by the oldtimers and newcomers having information about how each have performed previously in similar situations. Often newcomers enter groups with reputations known by the oldtimers and vice-versa [12]. One kind of reputation both oldtimers and newcomers may know is about each other’s previous performance on similar tasks. Kollock [43] suggests that performance history may be used as a signaling mechanism to facilitate efficient coordination in collective action dilemmas, and it decreases the costs of coordination among group members [44], [45]. Coordinative ability may improve from information because social uncertainty is reduced and trust is increased: the group members (new and old) know how the others behaved in the past and can therefore make more informed decisions about what behavior is necessary to achieve efficient coordination in the future.

**Hypothesis 4:** As the amount of information known by the group members about each other’s previous performance increases, coordination increases – such that groups that receive full information achieve more efficient coordination compared to when no information is provided; groups that receive full information achieve more efficient coordination compared to when partial information is provided, and groups that receive partial information achieve more efficient coordination compared to when no information is provided.

## Methods

### Ethics Statement

This study and its consent procedure were approved by the Chapman University’s Institutional Review Board. Participants provided written, informed consent to participate.

### The Weakest-Link Coordination Game

We test these hypotheses in a laboratory. Laboratory experiments allow for strong *internal validity* and high *psychological realism* while also enabling us to isolate the impact member change has on trust and subsequent behavior [46], [47]. We employ a weakest-link coordination game [48], in which efficient coordination is attained when all individuals in the group choose the option that maximizes group value, but individuals are exposed to private risk by attempting group coordination when others choose not to coordinate. Specifically, in the general form of a weakest-link game there are *n* participants, and each participant *i* chooses an integer *e_i_* between 1 and *ē*. The payoff of participant *i* depends on *e_i_* and the minimum integer chosen within the group Min (*e_i_*, *e_−i_*), i.e., *π_i_* (*e_i_*, *e_−i_*) = *a* Min (*e_i_*, *e_−i_*)−*b*|*e_i_* − Min (*e_i_*, *e_−i_*)|+*c*, where *b*|*e_i_* − Min (*e_i_*, *e_−i_*)| denotes the deviation cost and *a, b* and *c* are constants. Table 1 shows the weakest-link game used in the current study (*n* = 4, *ē* = 7, *a = *0.5, *b = *0.5 and *c = *3). Participants in a 4-person group could choose any integer *e_i_* between 1 and 7. From Table 1 we see that the set of outcomes where no one has an incentive to change their selected integer (or equilibria) is located along the diagonal. The Pareto-optimal (or best) equilibrium, however, that provides the highest payoffs to all participants, occurs when each participant chooses the highest integer, *ē* (the integer 7 in our studies).

**Table 1:** Payoffs in the Weakest-Link Coordination Game.

Your Choice		Minimum Value of X Chosen						
		7	6	5	4	3	2	1
	**7**	$6.50	$5.50	$4.50	$3.50	$2.50	$1.50	$0.50
	**6**		$6.00	$5.00	$4.00	$3.00	$2.00	$1.00
	**5**			$5.50	$4.50	$3.50	$2.50	$1.50
	**4**				$5.00	$4.00	$3.00	$2.00
	**3**					$4.50	$3.50	$2.50
	**2**						$4.00	$3.00
	**1**							$3.50

There are several benefits gained from using a weakest-link game. First, because free-riding is impossible, “cooperation [in the weakest-link game] … rests on trust” [49](p. 2), the game’s design isolates our primary mediating variable, trust [50]. Second, weakest-link games model many group tasks common in organizational settings. Consider three examples. A customer’s satisfaction with a hotel is often a function of the lowest quality of service received during their stay. Therefore how the “weakest” staff member serves the customer determines the overall performance of the group [29]. Air traffic control is another example: airplanes cannot take off until luggage is stored, passengers are seated, permission to take off is granted, and the plane is fueled [51], [52]. Supply chain alliances, who reduce their partner base to make each partner non-redundant [53], face a weakest-link game when launching new initiatives since each partner must provide a necessary component of the project or product [54]. Therefore, how the “weakest” employee or partner performs determines the overall performance of the airline or alliance.

### Experimental Design

One hundred and ninety-two students enrolled at a small, private university in the Western United States participated in exchange for a $7 show-up fee and an opportunity to receive additional money based on their decisions during the task. This sample was 40% male, with an average age of 20 years old, and 16% were graduate students. The current study used a one-way between-subjects design with 48 participants in each of the four conditions: a control condition and three newcomer conditions. In the control no-newcomer condition no newcomers were introduced during the task. The three newcomer conditions were as follows: newcomer/no-information condition (newcomers were placed within existing groups), newcomer/partial-information condition (oldtimers were aware of the newcomers’ previous performance), and newcomer/full-information condition (oldtimers and newcomers were aware of each other’s previous performance).

### Procedure, Task, and Conditions

Participants arrived to the laboratory in groups of 24, were forbidden to communicate, and were seated at individual computer terminals. All participants were provided with written instructions to the weakest-link game (available in Appendix S1) and were asked to follow along as the experimenter read the instructions out loud. These instructions highlighted that each person would be randomly assigned to a four-person group and play 10 periods in the weakest-link game and that their final earnings were a function of their group’s choices during the game. After the instructions were presented, participants could ask questions. We also conducted a short quiz to verify understanding of the instructions and the game.

The computerized experimental sessions used the software program z-Tree [55] to record participant decisions. Each session proceeded in two parts. In the first part, 24 participants were randomly assigned to a four-person group to play 10 periods of the weakest-link game. Participants stayed in the same group throughout all 10 periods. Although, participants knew the end period in the first part, they did not know about the second part of the experiment. This was necessary so that we could compare the pattern of group performance in the first 10 periods to those in previous research using weakest-link games.

At the beginning of each period, and based on a matrix provided (see Table 1), all participants were asked to enter their choice between 1 and 7. The value chosen by the participant and the minimum value chosen by all members in the group (including the participant) determined the payoff in any one period. Participants did not know the other participants’ choices before making their selection. The Pareto-optimal (or best) equilibrium that provides the highest payoffs to all participants occurs when each participant chooses the highest integer (the integer 7 in our studies). Thus, the greater the number chosen the greater the attempt of the individual to achieve group efficiency. After all participants made their decisions, the output screen displayed the minimum value between 1 through 7 chosen by group members, as well as the participant’s own payoff. Participants recorded their results in a hardcopy record sheet, and then moved on to the next period.

In the experiment’s second part, participants played the weakest-link game for another 10 periods. However, before the last 10 periods were played, one of four conditions occurred in the experimental session. Participants in the control condition were informed that they would play another 10 periods with the same group members. Participants in the newcomer conditions were informed that two members of their group would be randomly chosen by the computer and exchanged for two new participants from a different group. Thus, this new group was composed of two oldtimers (two members for the previous group) and two newcomers. Note that the current research design is such that when member change occurred, each group member perceived themselves and their remaining partner as oldtimers and the two new group members as newcomers. This group remained fixed for all 10 periods of the second part of the experiment. As explained to the participants, these two newcomers both came from the same group.

In the newcomer/no-information condition, oldtimers knew nothing of the newcomers’ previous performance and vice versa. In the newcomer/partial-information and newcomer/full-information conditions, participants received partial or full information about the performance of other participants, respectively. In particular, in the newcomer/partial-information condition, the computer randomly selected and informed two out of four group members (two newcomers or two oldtimers) about how the other two members performed in the first part of the experiment. The information displayed on the computer screen was about both participants’ choices in their group and the minimum group choice in each period. For example, a participant would see on the computer screen four columns: column one would be the periods listed from 1 through 10; column two provided what Player A (a newcomer) chose in each period; column three provided what Player B (the other newcomer from the same group as Player A) chose in each period; and column four showed the minimum value chosen by that group in each period. In the newcomer/full-information condition, the computer informed both the newcomers about the performance of oldtimers and oldtimers about the performance of newcomers. In other words, everyone had information about the prior performance of other group members before they began the second part of the experiment.

It should be noted that our design makes newcomers and oldtimers distinguishable to participants; participants can differentiate who is new in their group and who is not. From the participant’s perspective, the instructions inform them that they will be joined by two new group members: each participant is thus an oldtimer from their perspective. This design allows us to control for the amount of experience each participant had in their group – i.e., everyone experiences 10 periods of game play before member change occurred – thereby preventing our findings from being credited to changes in “role experience” among group members rather than member change [8].

After learning their group’s performance in period 10, but before the beginning of the experiment’s second part, all participants completed a survey questionnaire assessing their trust level towards other participants. Participants also completed a demographic questionnaire at the end of the second part of the experiment. After completing the entire experiment, participants received a total earnings sheet and the experimenter selected one period for payment from both the first and second parts of the experiment by rolling a 10-sided die twice in front of the group. Participants earned $18 on average, and sessions lasted approximately 45–50 minutes.

### Measures

The primary dependent measures in the current study include a behavioral measure of *coordination choice* and a survey measure for *trust*. *Coordination choice* is assessed at the individual level and measured on a scale from 1 to 7 [48]. Participants selected their decision (1–7) in each of the following periods: periods 1–9 and periods 11–19. Periods 10 and 20 were excluded in our analysis to avoid “endgame effects” [56] and to remain consistent with previous research using weakest-link games [20]; however their inclusion did not affect the significance of our hypothesis testing. Recall that the Pareto-optimal (or best) equilibrium that provides the highest payoffs to all participants occurs when each participant chooses the highest integer (the integer 7 in our studies). Thus, the greater the number chosen the greater the attempt of the individual to achieve group efficiency. We also assess how much an individual trusts that all of their fellow group members would select the value 7 (the highest coordination choice) in every period of the upcoming 10 periods. The *trust* scale is an index composed of 6-items adapted from Robinson [19] and showed high reliability (α = 0.91).

To further probe how trust in group members was affected by newcomers, several additional measures were included in the survey at the beginning of the second part of the experiment. Participants were asked to make non-incentivized *behavioral predictions* of what they believed each member of their group (i.e., Person A, Person B, and Person C) would select in the upcoming period. In the newcomer condition, participants were informed that Person A and B were the new group members and Person C was the remaining group member. All survey items are provided in Appendix S2.

Three variables were used as controls for the current study. First, considering that gender has been found to affect interdependent decision making in mixed-motive tasks, we recorded and coded a participant’s gender as 1 = male and 0 = female [57]. Second, because previous research has found that those with educational backgrounds in economics/business behave differently than other majors in mixed-motive settings [58], we coded each participant’s majors as 1 = economics/business major, 0 = otherwise. Lastly, we controlled for an individual’s *group performance history* as the average of group’s minimum chosen value across the periods in the previous game. This last control was necessary considering that behavioral norms often emerge through repeated interactions with interdependent others and these behaviors can “spillover” into future tasks [15], [59]. In addition, this control was necessary considering that the effect of newcomers on oldtimer’s perceptions and behavior can change as a function of how the oldtimers performed prior to newcomer arrivals [60]. All data from the current experiment are available upon request.

## Results

In testing our hypotheses and conducting post hoc analysis we use variations of ANOVA and panel regressions. When conducting various ANOVA analysis we mainly used the average across Periods 1–10 (or Periods 11–20) per subject as one independent observation. This is a standard practice in management and organization sciences. When appropriate, we also examined the robustness of our results using one group as one independent observation. Finally, we report the estimation results of random effect regressions, controlling for subject effects.

**Figure 1:**
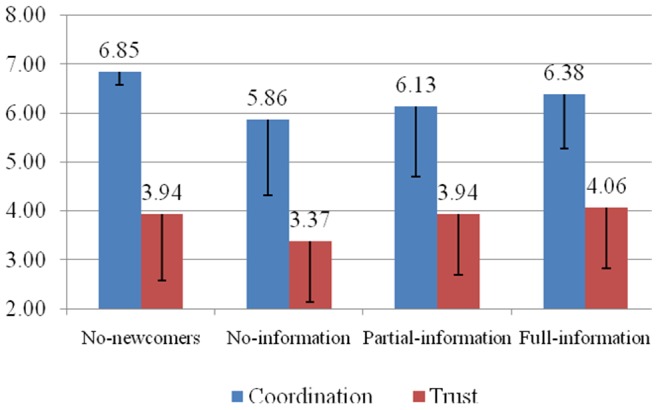
Mean Coordination Choice (in Periods 11–19) and Trust by Condition.

Figure 1 provides the mean *coordination choice* values (for periods 11–19) and *trust* levels for participants across all conditions. Gender and educational background did not have any significant effect on the outcome variables and are excluded from further analysis. A MANOVA, with *group performance history* as the independent variable and mean coordination choice across periods 11–19, trust, and predicted future behavior as dependent variables, found that *group performance history* was a positive predictor of coordination choice, trust, and predicted future behavior of oldtimers; all *F*s (1, 189) >6.87, all *p*s <0.01. However, the inclusion of *group performance history* did not affect the relationships among our constructs in our hypothesis testing: the presence of newcomers impacted a participant’s behavior and perceptions of others’ trustworthiness independent of their group performance history. Also, an ANOVA, with mean coordination choice as the dependent variable and condition as the independent variable, found that a participant’s mean coordination choice did not change as a function of condition in periods 1–9; *F* (3, 188) = 0.33, *p* = 0.80. This null finding is expected since the newcomer condition occurred after the end of period 10. Unless otherwise specified, all statistics provided in our analysis were reported using one-tailed tests and exclude all control variables; however, even with their inclusion, our effects remained significant at the 5-percent level.

### Hypothesis Testing

Hypothesis 1 posits that group members experiencing the presence of newcomers coordinate less efficiently compared to those in groups that do not experience the presence of newcomers. We test this hypothesis in two ways. First, we compare the mean *coordination choice* between the no-newcomer condition and the newcomer/no-information condition. In support of Hypothesis 1, an ANOVA, with mean coordination choice as the dependent variable and condition as the dependent variable, shows that individuals choose less efficient coordination values in groups with newcomers (M = 5.86, S.D. = 1.55) compared to those groups without newcomers (M = 6.85, S.D. = 0.27); *F* (1, 94) = 18.88, *p*<0.001, η^2^ = 0.17. Second, we employ a panel regression with subject specific random effects, where *coordination choice* in periods 11 through 19 is the dependent variable and the independent variables are group performance history (i.e., group coordination in periods 1 through 10) and a treatment dummy variable (1 = newcomer/no-information and 0 = no-newcomer). The estimation of the panel regression shows that, even when controlling for the history of play, a treatment variable is negative and significant; *β* = −1.41, *Z* = −4.91, p<0.01.

Hypothesis 2 posits that group members experiencing the presence of newcomers would trust their fellow group members less compared to when no newcomers were present. In support of Hypothesis 2, an ANOVA, with trust as the dependent variable and condition as the independent variable, shows that *trust* is lower for individuals in the newcomer/no-information condition (M = 3.37, S.D. = 1.23) compared to those in the no-newcomer condition (M = 3.95, S.D. = 1.37); *F* (1, 94) = 4.73, *p*<0.05, η^2^ = 0.05.

Hypothesis 3 posits that trust would mediate the negative relationship between the presence of newcomers and coordination in a group. We test for mediation following necessary steps outlined by Kenny, Kashy, and Bolger [61]. In step 1, a regression found a significant negative effect of the presence of newcomers on trust; *β* = −0.58, S.E. = 0.27, *p*<0.05. In step 2, a regression found a significant positive correlation between coordination and trust; *β* = 0.26, S.E. = 0.09, *p*<0.01. Finally, in step 3, a Sobel test found complete mediation of newcomers and *coordination* by *trust*; *Z* = −1.74, *p*<0.05. Therefore, there is support for Hypothesis 3.

Lastly, Hypothesis 4 posits that increasing the information known by the group members about each other’s previous performance would increase coordination. We test this hypothesis in two ways. First, we employ a panel regression with subject specific random effects, where *coordination choice* in periods 11 through 19 is the dependent variable and three ordinal newcomer conditions (1 = no information, 2 = partial information, and 3 = full information) is the explanatory variable. The estimation of the panel regression shows a positive linear relationship in the predicted direction; *β = *0.46, *Z* = 2.66, *p*<0.01. Next, we examine whether each additional set of information significantly improves coordination. We follow the procedure outlined by Winer [62] by comparing *coordination choice* in the newcomer/no-information condition (as a pseudo-control condition) to newcomer/partial-information and newcomer/full-information conditions (p. 89). A Dunnett *t*-test (*k* = 3, *n* = 48) finds a significant difference in mean *coordination choice* between the newcomer/full-information (M = 6.38) and the newcomer/no-information condition (M = 5.86); *t*_D_ (141) =  −1.85, *p*<0.05; but no significant difference in mean *coordination choice* between newcomer/full-information (M = 6.38) and newcomer/partial-information conditions (M = 6.13); *t*_D_ (141)* = * −0.89, *p* = 0.19. Additional analysis finds that there is also no difference in mean *coordination choice* between the newcomer/no-information and newcomer/partial-information conditions. Therefore, there is partial support for Hypothesis 4: full information negates the negative effect of newcomers on coordination, while partial information does not.

### Alternative Explanations of Primary Results

In considering previous empirical work [16], task flexibility and group learning are potential alternative explanations for our findings. Group tasks are considered flexible when group members may “fill in” for each other in the group to maintain high performance [63]. The weakest-link game structure leaves no room for a participant to replace the choices of other group members: *everyone* must choose 7 for the group to perform at the highest efficiency. The inflexibility of the weakest-link game, which is constant in both conditions, removes task flexibility as an alternative explanation.

To consider the group-learning explanation, Van der Vegt and colleagues [16] conclude that “any amount of [member change] may create an uncertain interpersonal environment in which team members are uncomfortable taking the risks necessary to engage in learning behaviors” to improve group performance (p. 1186). While we cannot assess group learning directly, we can examine whether risk-taking explains our findings. We did so by examining the proportion of individuals selecting a coordination value above the minimum value chosen in their group (thus indicating high risk-taking) [51], and whether such risk-taking occurred among a lower proportion of individuals in the newcomer condition compared to the control condition (p. 104). In isolating periods 12 through 19, we coded an individual’s coordination-value choice in each period as a 1 if it was above the group’s minimum choice in the previous period and 0 otherwise. Across periods, all cases where the group’s minimum value was 7 were excluded from the analysis because participants could in no way take a risk so as to improve group performance. The final ratios of risk-taking behavior to all actions taken in the no-newcomer condition and no-information, newcomer condition were 45/88 and 105/188, respectively. Using generalized estimating equations with period as the within-subject factor [64], no significant difference in risk-taking between no-newcomer condition (M_ = _48.9%) and newcomer/no-information condition (M = 55.9%) was found; *β* = 0.28, χ^2^ (1) = 1.17, *p*>0.20.

### Post Hoc Analysis: Trust of Newcomers and Oldtimers

**Figure 2:**
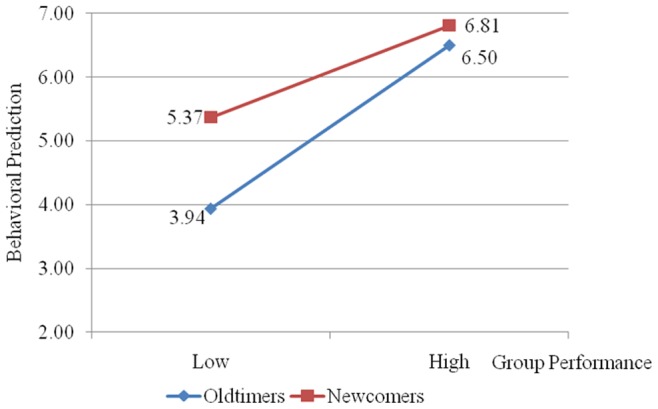
Behavioral Prediction Based on Group Performance History in the Newcomer/No-Information Condition.

While our primary findings support our hypothesis that trust mediates the relationship between newcomers and group performance, a remaining question is *who in the group was not being trusted when newcomers arrived*? In other words, which subgroup – oldtimers and/or newcomers – is driving distrustful behavior? To address this question, we first observe that an individual’s *behavioral predictions* for newcomers and oldtimers are significantly correlated with their subsequent *coordination choice* in period 11; both *r*s >0.40, both *p*s <0.001. This finding, combined with our finding that *Trust* mediates the relationship between newcomers and *coordination choice* (Hypothesis 3), leads us to assume that these behavioral predictions are representations of trust. We next compare an individual’s *behavioral prediction* for the oldtimer’s behavior to a newcomer’s behavior in the newcomer/no-information condition as a function of the group’s performance history in periods 1–9. We coded a group as high performing if that group’s average integer selected across the first nine periods was 7, and as low performing otherwise. The results are displayed in Figure 2. A repeated-measure ANOVA, with *subgroup* (1 = oldtimer and 0 = newcomer) as a within-subject factor and *group performance history* as a between-subject factor finds that participants believed the newcomers would choose a higher value in period 11 (M = 5.86, S.D. = 1.50) compared to the oldtimer (M = 4.81, S.D. = 1.90); F(1, 45) = 11.96, p<0.01, η^2^ = 0.21, and this main effect is qualified by a significant interaction: only when group performance is low do participants predict that newcomers will coordinate more efficiently than oldtimers; F(1, 45) = 4.94, p<0.05, η^2^ = 0.10. Considering there was no way for participants to delineate between the two newcomers (Person A and Person B), we averaged these best guesses and compared this average to the best guess for Person C (the oldtimer). A Pearson correlation supported this decision for averaging: the correlation of best guesses of value choice between Person A and Person B was *r* = 0.93, *p*<0.001.

The group identity literature on the “black-sheep effect” may explain this negativity toward oldtimers [65]. The black-sheep effect occurs when group members react to non-cooperative (or poor performing) in-group members more negatively than those not part of the group [66]. Two common reactions to black sheep are greater distrust and subsequent defensive behavior against the black sheep in future interactions [67]. In relation to our findings, an oldtimer of a group experiencing poor coordination during periods 1–9 may view the remaining oldtimer more negatively (and distrust them more) compared to incoming newcomers; but this is only the case where *Group Performance History* is poor.

## Discussion and Conclusion

In summary, our results support our hypotheses. Newcomers negatively affect a group’s ability to coordinate efficiently because trust declines among the group members. However, this finding is qualified by additional analysis that suggests oldtimers trust each other less compared to the newcomers when group performance history is poor. This finding is consistent with the black-sheep effect from the social psychology literature. We also find that information about group members’ previous performance mitigates the negative effect that newcomers have on coordination. However, whereas full information bridged the gap caused by newcomers, partial information did not. Trust mediates the relationship between the provision of information about group members and the coordination patterns observed, such that greater coordination is found in groups with greater information because such information increases levels of trust in the group.

Our investigation of how and why newcomers affect group performance contributes to our understanding of group dynamics in several ways. First, previous work on group member change has encouraged scholars to investigate how group emergent states mediate newcomer effects on group performance [13]. Some work has surfaced recently to address this question. Specifically, research on self-managing manufacturing teams has found that team turnover (i.e., the number of individuals leaving the team divided by group size) negatively impacts team performance through the emergent states of reduced team learning and task flexibility [16]. Our laboratory research complements this field research by identifying group trust as an additional emergent state that explains the negative effect of member change on group performance: newcomers reduce levels of trust in groups, and, in turn, trust negatively affects how efficiently the group coordinates. Thus, whereas previous research has identified how the developed in-group processes of a group (e.g., flexibility) mediate the newcomer-performance relationship [16], the current paper highlights how the quality of interpersonal relationships (e.g., trust) also explains the member change-group performance relationship.

In addition to showing that trust mediates the relationship between member change and group performance, our supplemental findings suggest that oldtimers’ trust levels for one another are actually most directly affected when newcomers enter groups. Specifically, as would be predicted by the “black-sheep effect,” newcomers create distrust in poor performing groups, but this distrust is about how oldtimers expect each other to behave, rather than about how the newcomers will behave [65]. This finding begins to address the call to understand how trust and behavior among oldtimers change when newcomers arrive [12] and suggests that managers and leaders of collective action need not only worry about how oldtimers perceive the newcomers but how the oldtimers perceive each other – especially when the group is performing poorly prior to newcomers arriving. Indeed, most of the applied research on organizational change and development focuses on how managers may help newcomers respond and adapt effectively to existing groups [68]. Whereas socializing newcomers to new work environments is necessary to improve their transition, our research suggests that existing norms and relationships among oldtimers are not immune to change. Specifically, managers should not just help socialize newcomers to oldtimers, but oldtimers to oldtimers when member change happens.

A third implication of the current research is with respect to navigating the negative effects of newcomers on group performance. Our study finds that information is critical in mitigating the negative effects of newcomers; however, only when information is known by both oldtimers and newcomers. This finding of information as a mechanism for alleviating social uncertainty also has implication for collective action research. For over a decade, scholarship has asked how individuals may signal intent and commitment to cooperate with others without traditional mechanisms such as group discussion and promise making [43]. Our research suggests that information about prior performance may be a useful signal of intent and commitment to cooperate. Filling this gap has both managerial and theoretical importance considering that some collective efforts are structured in ways that prevent group discussion – such as situations where group members are geographically distributed (e.g., virtual teams) or newcomers and oldtimers speak different languages. For example, in the forests of New Brunswick in the late 1800s, several Native American tribes, who had cooperatively maintained the moose and caribou population for centuries by conservative hunting, abruptly ceased cooperating and annihilated these invaluable food sources. These tribes’ deliberate actions occurred soon after the arrival of white, French settlers to the region [10]. It is possible that information about prior performance and intent shared between the oldtimers and the newcomers may have mitigated this effect. Indeed, the current paper shows how the “shadow of the past” (i.e., information about past performance) can be a signal of both intent and experience [44]; however, this finding is qualified by the observation that only when everyone shares information do the negative effects of newcomers decrease.

The findings in the current paper raise several new directions for future examination. First, our experiments used a game that requires everyone to cooperate to achieve the best collective outcome. Some collective action problems are not so strict, but rather allow group members to cover for each other when one member does not do what is best for the group [69]. For instance, before launching a generic advertising campaign, some industries only need a portion of the total population to chip in [70]. Free riding is possible but so is the ability to make up for free riders. Future research may examine how thresholds associated with achieving collective action interact with the negative effect of newcomers on coordination. It may be that the negative effect of newcomers decreases as the ability to cover for other’s mistakes increases. This may be because social uncertainty and trust are no longer of great concern since everyone is not needed to pull through. In addition, newcomers may be welcomed by oldtimers in some cases compared to others depending on whether the value of collective action is certain to be worth the effort [71]. For instance, should they be uncertain that collective action will produce benefits that will surpass the cost, oldtimers may strategically welcome newcomers into the fold in hope to use these newcomers’ resources before expending their own.

A second avenue for future research is in regard to group size. Our study used four-person groups, compared to many collective actions that involve dozens or hundreds of individuals. While we know that large groups can achieve collective action when allowed to grow gradually over time [20], future research may ask how group size interplays with the presence of newcomers. Indeed, simulation research has found that one newcomer (or a few) has little effect on cooperative routines among very large groups [72]. It may be that, as the proportion of oldtimers to newcomers decreases, the negative effects of newcomers increases.

Lastly, the negative, linear coordination trends found in all three newcomer conditions leave a question: what mechanisms alter the negative direction of the group coordination caused by newcomers? Communication may be one mechanism. Relationship repair research reminds us that the purpose of the communication (e.g., communicating intent, apologies, and making penance) is just as critical as allowing communication [73]. Considering that individuals often require additional penance when promises are broken [74], [75], future research may investigate whether newcomers and oldtimers require different means of amends depending on who is communicating with whom.

Jesus is recorded to have said that “… no man putteth new wine into old bottles; else the new wine will burst the bottles, and be spilled, and the bottles shall perish” (Luke 5∶37, KJV). In line with the above saying, we found that newcomers burst the group’s ability to coordinate efficiently – apparently because the oldtimers lost trust in one another when newcomers were present – resulting in spilled potential value. However, information can be a signaling mechanism reducing the negative shock of newcomers on groups.

## Supporting Information

Appendix S1 **Study Materials.**
(DOCX)

Appendix S2 **Survey Items.**
(DOCX)
