# Supplementary material for: You Can’t Put Old Wine in New Bottles: The Effect of Newcomers on Coordination in Groups
Source: PLoS One. 2013 Jan 30;8(1):e55058. doi: 10.1371/journal.pone.0055058 (PMC3559334; doi:10.1371/journal.pone.0055058)
Supplement: Appendix S1 — Study Materials. (DOCX) [file pone.0055058.s001.docx]

**Instructions – Part 1**

In this experiment you will participate in a game with three other participants. You will not know the identity of the participants you are grouped with. The experiment will consist of 10 periods and in all 10 periods you will participate with the same participants.

One period will be randomly selected for payment at the end of the experiment. After you have completed all periods a token will be randomly drawn out of a bingo cage containing tokens numbered from 1 to 10. The token number determines which period is going to be paid.

In each period, you will select a number denoted by X. The values of X you may choose are 1, 2, 3, 4, 5, 6, or 7. When you are ready to make your decision, click on the “input box” below “Enter your choice of X” and the program will allow you to enter in your number choice. The value you pick for X and the minimum value of X chosen by all members in your group (including yourself) will determine your payoff in any one period. When you are finished making your choice, click “Submit”.

**[The table presented in Table 1 was provided here]**

Table 1 tells you how you earn money. Please look at the table now. The entries in the table give each participant’s Experimental Dollar earnings from selecting alternative values of X. The earnings in each period may be found by looking across from the value you choose on the left-hand side of the table, and down from the minimum value chosen from the top of the table. For example, if you chose a 4 and the minimum value chosen by all members in your group was a 3, you earn $4.00 that period. Alternatively, if you chose 4 and the minimum value of X chosen in your group was 4, then you earn $5.00. Note that all four participants (including you) have the same payoff table.

The experiment will consist of 10 periods, where in each period you will be grouped with the same three participants. In each period the following will occur:

1. At the beginning of the period, you are asked to enter your choice of X for that period. Your choice of X is private and should not be discussed with anyone during the experiment. Note that you do not know the other participants’ choices of X before making your selection.

2. After all participants make their decisions, the computer will determine the minimum value of X chosen in your group and display it on the output screen.

3. Then the computer will determine your earnings (you may confirm this using Table 1) for that period. Please record your results for the period on your record sheet under the appropriate heading.

**General Instructions - Part 2 (sample)**

Considering that we have some time remaining in this session, we will play this same game for another 10 rounds.

[C*ontrol condition:* You will be with the same people that were in your group in the previous game.]

*[Added the following before any newcomer condition]* The only difference will be that the computer will randomly select two players from each group and switch them with two players from another group.]

[*No information condition*: Also, for this second game, you, your remaining group members from the first game, and the new members will not be provided information about how the others performed in the previous game. So, you (and the group member from your first round) will not know how the two new members performed in their previous game. And, the two new members will not know how you two performed in the previous game.]
