# Supplementary material for: You Can’t Put Old Wine in New Bottles: The Effect of Newcomers on Coordination in Groups
Source: PLoS One. 2013 Jan 30;8(1):e55058. doi: 10.1371/journal.pone.0055058 (PMC3559334; doi:10.1371/journal.pone.0055058)
Supplement: Appendix S2 — Survey Items. (DOCX) [file pone.0055058.s002.docx]

**Trust scale items (1 = strongly disagree; 6 = strongly agree)**

1. I expect all three members of my group to treat me in a predictable fashion by selecting the number 7 in each of the upcoming games.
2. I believe all three of the members in my group have high integrity.
3. I think all three members in my group will treat me fairly by selecting the number 7 in each of the upcoming games.
4. I believe all three members in my group have good motives and intentions.
5. I think all three members will continue to work well together by selecting the number 7 in each of the upcoming games.
6. I fully trust all three members in my group to select the number 7 in each round of the upcoming games.

**Behavioral Prediction sample item**

Please state your prediction (your “best guess”) or what each of the other group members will choose in the upcoming round.

Player (A) 1 2 3 4 5 6 7
